# Cognitive Improvement in Methamphetamine-Dependent Males: A Randomized Trial Comparing Different Exercise Interventions with Behavioral and fNIRS Evidence

**DOI:** 10.3390/brainsci16050451

**Published:** 2026-04-24

**Authors:** Xuejie Zhang, Jisheng Xu, Xulin Zhou, Yan Yang, Guosi Ying, Xue Li

**Affiliations:** School of Sports Medicine and Health, Chengdu Sport University, Chengdu 641418, China; zhangxuejie0411@163.com (X.Z.);

**Keywords:** methamphetamine, fNIRS, exercise, cognition

## Abstract

**Background**: Long-term methamphetamine use disrupts brain function and impairs cognition. Currently, there is a lack of effective treatments for cognitive dysfunction in this population. This study aimed to investigate the effects of different exercise interventions on cognitive function and brain activation in methamphetamine-dependent individuals and to explore the potential neural mechanisms underlying cognitive improvement. **Methods**: In this randomized, assessor-blind, controlled study, 162 male methamphetamine-dependent individuals in compulsory isolation were randomly assigned to one of four groups: traditional regimen training (TR, *n* = 41), aerobic exercise (AE, *n* = 40), multimodal cognitive exercise training (MC, *n* = 40), and a control group (MA, *n* = 41). All participants completed an 8-week intervention. Cognitive function was assessed before and after the intervention using the Stroop task, while fNIRS was used to measure task-related hemodynamic responses. In addition, the Memory and Executive Screening (MES) and choice reaction time tests were used to evaluate cognitive and psychomotor performance. **Results**: After 8 weeks, traditional regimen training (*p* = 0.006), aerobic exercise (*p* = 0.024), and multimodal cognitive exercise training (*p* < 0.001) all significantly improved Stroop task accuracy. Aerobic exercise significantly increased activation in L-DLPFC (*p* = 0.044), R-DLPFC (*p* = 0.036), and L-FPA (*p* = 0.038), improved MES-T scores (*p* < 0.001) and shortened choice reaction time (*p* < 0.001). Traditional regimen training increased L-DLPFC activation (*p* = 0.026), improved MES-T scores (*p* < 0.001), and shortened choice reaction time (*p* < 0.001). Multimodal cognitive exercise training increased activation in L-DLPFC (*p* = 0.006), R-DLPFC (*p* = 0.014), and L-FPA (*p* = 0.002), improved MES-T scores (*p* < 0.001) and shortened choice reaction time (*p* < 0.001). **Conclusions**: Cognitive impairment in methamphetamine-dependent individuals may be associated with reduced prefrontal functional activity. Different exercise modalities produced different patterns of cognitive improvement and brain activation, with multimodal cognitive exercise training showing the largest overall benefit.

## 1. Introduction

The World Drug Report 2024 highlights the worsening global drug problem due to emerging drug types and unprecedented supply and demand. In 2022, approximately 292 million people worldwide used drugs, a 20% increase over the past decade [1]. Methamphetamine addiction has therefore become a critical global public health issue. Also known as “ice”, methamphetamine is chemically derived from ephedrine [2].

Studies suggest that among multiple substance users, long-term use of methamphetamine can lead to impairments in memory, attention, information processing, and executive function [3,4], with its impact on working memory being the most severe [5]. Even after cessation, cognitive impairments may persist [6]. These impairments are associated with prefrontal dysfunction [7,8], nerve terminal damage [9], and hippocampal and striatal dysfunction [3,10].

In recent years, non-pharmacological interventions have garnered considerable attention as adjuncts to conventional addiction treatment [11,12,13,14,15,16,17,18]. Among these, physical exercise has demonstrated promising benefits for both physical and cognitive function in individuals with substance use disorders [19,20]. Aerobic exercise, for example, has been shown to enhance executive control and restore cognitive performance by increasing prefrontal cortex activity and strengthening brain network connectivity [14,15,17]. Other forms of rehabilitation exercise may improve inhibitory control and positively influence cognitive outcomes through both neurophysiological and behavioral pathways [12,16,18]. However, most existing research has focused on unimodal exercise interventions, often with small samples, limited durations, or a lack of neuroimaging evidence to clarify mechanisms.

Emerging evidence suggests that combining physical exercise with cognitive tasks—known as multimodal or dual-task training—may further enhance neural plasticity and yield greater cognitive benefits than exercise alone [21,22]. While commonly used for mild cognitive impairment, dementia, and stroke [23], multimodal training may have synergistic effects beyond exercise or cognitive training alone [24,25,26]. However, its application in methamphetamine-dependent individuals remains largely unexplored. Critical questions thus remain: Are multimodal exercise interventions superior to unimodal approaches for improving cognitive outcomes in this population? What are the underlying neural mechanisms?

Functional near-infrared spectroscopy (fNIRS) is a non-invasive neuroimaging technique that measures changes in oxygenated and deoxygenated hemoglobin in the brain. Based on the principles of neurovascular coupling and optical spectroscopy, fNIRS can indirectly assess neuronal activation [27]. This technique can help identify brain activation patterns and potential neural mechanisms through which exercise interventions influence cognitive outcomes in substance-dependent populations [28,29].

Although previous studies have suggested that exercise may improve cognitive function in methamphetamine-dependent individuals, most have focused on a single exercise modality. Direct comparisons among different exercise interventions remain limited, particularly in studies combining behavioral outcomes with neuroimaging measures. Accordingly, the present study aimed to address these gaps by comparing the effects of three distinct exercise modalities—traditional regimen training, aerobic exercise, and multimodal cognitive exercise training—on cognitive performance and prefrontal cortical activation in methamphetamine-dependent males. Using behavioral assessments and fNIRS before and after the 8-week intervention, we examined changes in cognitive performance and prefrontal cortical activation associated with each exercise modality. We hypothesized that multimodal cognitive exercise training would produce the greatest improvements in executive function and prefrontal activation compared with traditional regimen training and aerobic exercise, thereby providing insight into evidence-based rehabilitation strategies for cognitive dysfunction in substance use disorders.

## 2. Materials and Methods

### 2.1. Study Design and Participants

This study employed a randomized controlled, assessor-blind design. Between 31 January and 3 February 2024, 164 methamphetamine-dependent males were recruited from the Compulsory Isolation Drug Rehabilitation Center in Sichuan Province under strict inclusion and exclusion criteria. The study was approved by the Ethics Committee (Approval No. 202406) and complied with the Declaration of Helsinki. All participants provided written informed consent. The trial was registered with the Chinese Clinical Trial Registry (ChiCTR2400080819).

**Inclusion and exclusion criteria:**

**Inclusion criteria:** ① A meeting DSM-V criteria for methamphetamine substance dependence, with a drug use history exceeding one year; ② Right-handed (dominant hand); ③ Educational level of primary school or above; ④ Deemed eligible based on exercise risk assessment; ⑤ Able to commit to a rehabilitation period of at least two months; ⑥ Currently undergoing compulsory isolation and withdrawal for no more than six months; ⑦Voluntarily participates and provides written informed consent.

**Exclusion criteria:** ① Limb disabilities or physical dysfunctions; ② Infectious diseases (e.g., hepatitis, HIV) or severe, unhealed trauma; ③ Severe mental illnesses (such as schizophrenia, mania, mental retardation, etc.); ④ Severe organic diseases, family history of mental illness, or history of head injury; ⑤ Dependence on illicit drugs other than methamphetamine.

### 2.2. Randomization and Blinding

A priori power analysis was conducted using G*Power version 3.1 to determine the appropriate sample size for this study. Based on a repeated-measures ANOVA design with four groups and two measurement points, an effect size of 0.25, a significance level of 0.05, and a statistical power of 0.95, the required total sample size was estimated to be 76 participants. Considering a potential dropout rate of approximately 20%, as well as the substantial inter-individual variability commonly observed in cognitive outcomes, we increased the recruitment target accordingly. Ultimately, 164 participants were enrolled (approximately 41 per group), which ensured adequate statistical power to detect meaningful intervention effects.

Randomization was performed by an independent researcher who was not involved in participant recruitment, intervention delivery, or outcome assessment. Using the random number generator in Microsoft Excel, a random number was assigned to each of the 164 participants. Participants were then sorted in ascending order according to the generated random numbers and allocated sequentially to one of four groups in a 1:1:1:1 ratio: control group (MA, *n* = 41), traditional regimen training group (TR, *n* = 41), aerobic exercise group (AE, *n* = 41), and multimodal cognitive exercise training group (MC, *n* = 41).

**Figure 1:**
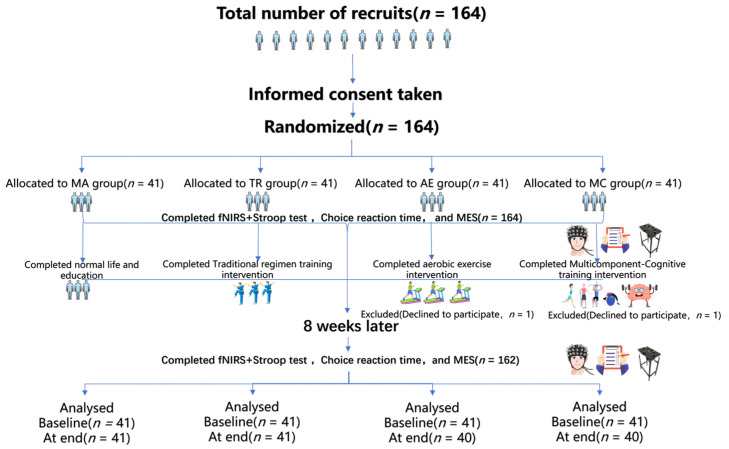
Flow diagram of participant recruitment, group allocation, intervention, and analysis in the 8-week randomized controlled trial.

This study used an assessor-blind design. All behavioral and neuroimaging assessments were performed by trained personnel who remained unaware of group allocation throughout data collection and analysis. Because of the nature of the exercise interventions, participants could not be fully blinded to treatment assignment. However, they were not informed of the specific study hypotheses or the expected comparative effects of the different interventions. In particular, participants in the control group were told that they would continue to receive standard institutional care, including routine health education and a structured daily schedule, but were not explicitly informed that they served as the no-exercise comparison group, thereby reducing potential expectancy bias. During the 8-week intervention, one participant in the AE group and one participant in the MC group withdrew voluntarily, yielding a final analytic sample of 162 (Figure 1).

### 2.3. Exercise Intervention

Based on previous studies demonstrating that long-term interventions and moderate exercise intensity significantly improve the cognitive function of methamphetamine-dependent individuals [30,31,32], the TR, AE, and MC groups in the present study participated in moderate-intensity exercise interventions. The intervention period lasted eight weeks, with a frequency of five sessions per week, and each session lasted one hour. All exercise sessions were supervised by trained instructors, and exercise intensity was monitored in real time using Bluetooth heart rate straps (Polar OH1, Polar Electro, Beijing, China) to maintain moderate intensity (60–70% of age-predicted maximum heart rate). Attendance was recorded throughout the intervention to monitor adherence. Throughout the intervention period, all participants received routine care, including diet and sleep guidance, while the MA group maintained its usual activity, lifestyle, and educational schedule.

**Traditional Regimen Training (TR):**

The traditional regimen training included 5–10 min of warm-up exercises, 45 min of formal training, and 5–10 min of relaxation exercises. The training consisted of Tai Chi and Pigua martial arts rehabilitation exercises, a comprehensive martial arts routine combining multiple styles, such as Pigua, Xingyi, Tai Chi, and Bagua.

**Aerobic Exercise (AE):**

The aerobic exercise training included 5–10 min of warm-up exercises, 45 min of formal training on power bicycles or treadmills, and 5–10 min of relaxation exercises.

**Multimodal Cognitive Exercise Training (MC):**

Multimodal cognitive exercise training integrated physical exercise with specific cognitive tasks, such as mathematical calculations, reverse repetition of words or numbers, and hand-eye coordination tasks using Swiss balls and foam balls. Participants were required to perform cognitive tasks concurrently with exercise training. Each session consisted of 5–10 min of warm-up exercises, 10–15 min of aerobic exercise, 10–15 min of strength training, 10–15 min of balance and coordination training, and 10 min of flexibility training, with cognitive tasks embedded during exercise.

### 2.4. Outcomes

In this study, the primary outcomes included Stroop task accuracy, reaction time, and task-related hemodynamic changes. Secondary outcomes comprised MES scale scores, choice reaction time, and the correlation between cognitive performance and brain activation levels.

#### 2.4.1. Stroop Task Detection

This study employed the classic color-word Stroop task to assess cognitive function, implemented using E-Prime 3.0. Participants were instructed to respond as quickly and accurately as possible to the stimulus color, with responses recorded within 1500 ms and an inter-trial interval of 5000 ms. The stimuli consisted of three words (“red”, “yellow”, “blue”), each displayed in one of the three colors, resulting in three congruent conditions (e.g., “red” in red) and six incongruent conditions (e.g., “red” in blue).

**Figure 2:**
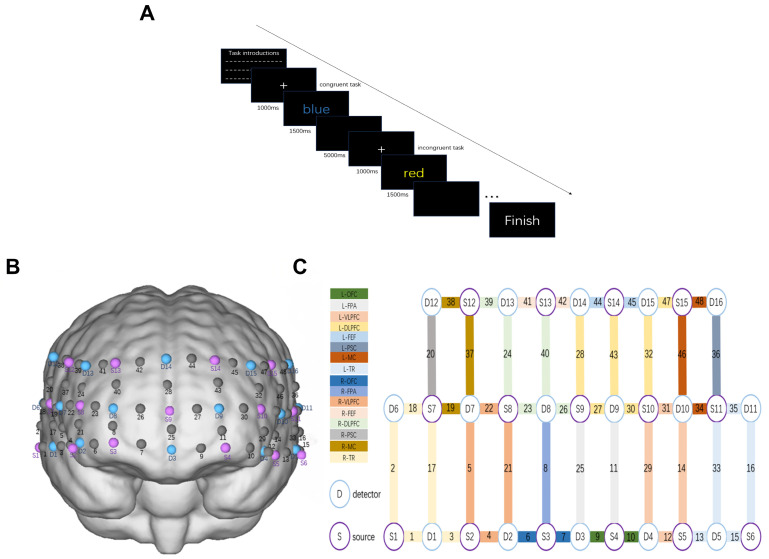
Experimental paradigm and fNIRS optode configuration. (**A**) Schematic of the Stroop task paradigm used in the experiment. The task includes both congruent (e.g., the word “blue” displayed in blue) and incongruent (e.g., the word “red” displayed in yellow) trials, each preceded by a fixation cross (1000 ms) and followed by a stimulus display (1500 ms). Participants responded to the font color, not the word meaning. (**B**) 3D representation of the fNIRS optode placement over the prefrontal cortex. Purple circles represent sources (S), blue circles represent detectors (D), and their pairings form measurement channels. (**C**) Channel layout and corresponding brain regions of interest (ROIs). Each numbered channel corresponds to a specific combination of source and detector, mapped onto regions including the left and right dorsolateral prefrontal cortex (L-DLPFC, R-DLPFC), ventrolateral prefrontal cortex (L-VLPFC, R-VLPFC), frontopolar area (L-FPA, R-FPA), orbitofrontal cortex (L-OFC, R-OFC), frontal eye field (L-FEF, R-FEF), motor cortex (L-MC, R-MC), primary somatosensory cortex (L-PSC, R-PSC), and temporal region (L-TR, R-TR). Color-coded boxes indicate ROI assignments.

The task included 12 practice trials (randomly presenting 6 congruent and 6 incongruent stimuli) followed by 24 formal trials (randomly presenting 12 congruent and 12 incongruent stimuli). Both reaction time and accuracy were recorded as outcome measures. Reaction time was defined as the time taken to respond to the stimulus, with shorter reaction times indicating better cognitive performance. Similarly, higher accuracy rates reflected superior cognitive function (Figure 2A).

#### 2.4.2. fNIRS Detection Procedure

This study employed the NirSmart-6000A (Danyang Huichuang Medical Equipment Co., Ltd., Danyang, China) to continuously measure oxygenated, deoxygenated, and total hemoglobin concentrations in the brain during task performance. The setup included 15 light source probes and 16 detectors, forming effective channels with an average transmitter-detector distance of 3 cm (range: 2.7–3.3 cm). According to Korbinian Brodmann’s brain parcellation method [33], this study focused on observing the prefrontal cortex and temporal lobe [34]. A total of 16 regions of interest (ROI) were divided (Figure 2B,C). Time-series data of oxygenated hemoglobin concentration changes were extracted for each channel and averaged across channels belonging to the same ROI to reflect regional cortical activation.

#### 2.4.3. The Memory and Executive Screening

The Memory and Executive Screening Scale (MES) is currently a widely used cognitive assessment scale for detecting mild cognitive impairment [35]. The scale is convenient to operate, takes a short time, is less affected by educational level, and can quickly understand the degree of impairment in major cognitive domains without obvious ceiling and floor effects [36]. The MES consists of two subtests: memory ability and executive function, with their respective scores recorded as MES-5R and MES-EX (50 points each). The total score of the MES scale (MES-T) is 100 points. A total score below 75 indicates mild cognitive impairment [35].

#### 2.4.4. Choice Reaction Time

Participants were instructed to extend the fingers of one hand and press the “start” key with the distal phalanx of the middle finger. When a signal key emits a signal, the participant presses the “signal” key with the same hand as quickly as possible, then presses the “start” key again to wait for the next signal. Each test requires completing five signal responses. The test concludes when all signal keys emit simultaneous sound and light signals, and the display screen shows the test result. The test is conducted twice, and the minimum value is recorded. The unit of measurement is seconds (s), with results rounded to two decimal places.

### 2.5. Data Processing

#### 2.5.1. Stroop Task Data

Stroop task data were analyzed and processed. Responses exceeding 1500 ms or below 150 ms were excluded as errors. The mean reaction time and accuracy rate for each participant were then calculated. Processed data were imported into Excel for further analysis, with higher accuracy and shorter reaction times indicating superior cognitive function.

#### 2.5.2. fNIRS Data Analysis

Data were processed using NirSpark 1.8.1. with the following steps: (1) unsatisfactory time intervals containing sudden, obvious, and discontinuous noise were excluded; (2) artifacts caused by motion and environmental interference were corrected (threshold standard deviation = 6.0; threshold amplitude = 0.5); (3) the raw data were filtered using a bandpass filter with a frequency range of 0.01–0.2 Hz; (4) the modified Beer–Lambert law was applied to calculate changes in oxygenated hemoglobin (ΔHbO), deoxygenated hemoglobin (ΔHbR), and total hemoglobin (ΔHbT). Because ΔHbO signals are more sensitive to task stimulation and exhibit a relatively high signal-to-noise ratio, ΔHbO was selected for analysis [37]. Based on the principle of neurovascular coupling, neural activity in the brain is inferred to coincide with increased ΔHbO in local brain regions. Thus, ΔHbO data were used to analyze hemodynamic changes in specific brain regions; (5) ΔHbO values from all Stroop task trials were averaged to obtain the mean ΔHbO response for each sampling point during the 2.5 s interval from stimulus onset to response completion in each channel; and (6) ΔHbO data from one or more channels within each region of interest (ROI) were averaged to represent task-related hemodynamic changes in the ROI [38].

#### 2.5.3. Data of MES Scale and Choice Reaction Time

The MES scale measured memory (MES-5R) and executive function (MES-EX), with a total score (MES-T) of 100. Scores below 75 indicated mild cognitive impairment. Additionally, the completion times for the choice reaction time task were statistically analyzed and sorted. Shorter reaction times were considered indicative of better cognitive function.

### 2.6. Statistical Analysis

Data were analyzed using SPSS 29.0. Normality and homogeneity of variance were assessed before formal analyses. Data conforming to a normal distribution were expressed as mean ± standard deviation (M ± SD). For data not meeting normality assumptions, the Friedman test was applied, with post hoc Bonferroni corrections used for multiple comparisons. A repeated measures analysis of variance (ANOVA) was conducted to evaluate the reaction time and accuracy rate of the Stroop task, fNIRS data, MES scale scores, and choice reaction time across four groups (TR, AE, MC, MA) × two test times (pre-test and post-test). When a significant interaction effect was observed, a simple effects analysis was performed. For the fNIRS data, false discovery rate (FDR) correction was applied to the *p*-values for the time × group interaction effects across the 16 ROIs. Simple effects analyses were performed only for ROIs that remained significant after FDR correction. Pearson correlation analysis was conducted to examine the relationships between Stroop reaction time, accuracy rate, brain activation levels, MES-T scores, and choice reaction time, with *p*-values also corrected using the Benjamini–Hochberg false discovery rate (FDR) method. A two-tailed *p* value < 0.05 was considered statistically significant.

## 3. Results

### 3.1. Participants’ Characteristics

The demographic and clinical characteristics of the four groups are presented in Table 1. Statistical analyses showed no significant baseline differences among the groups (*p* > 0.05). Mean heart rate during the intervention was also compared across the three exercise groups (TR, AE, and MC). No significant between-group difference was observed (*p* > 0.05), suggesting broadly comparable exercise intensity across the intervention groups.

### 3.2. Stroop Task Performance Before and After Exercise

**Figure 3:**
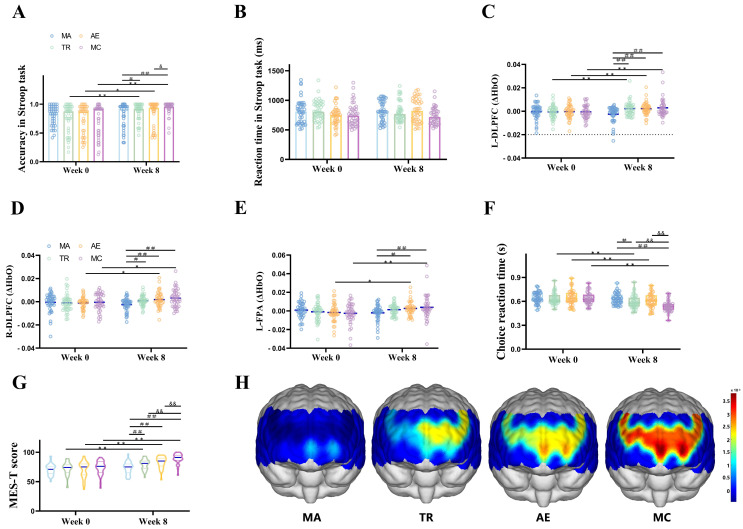
Results before and after the 8-week intervention. (**A**) Stroop task accuracy at Week 0 and Week 8. Bars represent group means, with overlaid individual data points. (**B**) Stroop task reaction time at Week 0 and Week 8. Bars represent mean response time, with dots showing individual performance. (**C**–**E**) Violin plots show task-related ΔHbO changes in three brain regions: (**C**) left dorsolateral prefrontal cortex (L-DLPFC), (**D**) right dorsolateral prefrontal cortex (R-DLPFC), and (**E**) left frontopolar area (L-FPA). (**F**) MES-T scores before and after the intervention. (**G**) Choice reaction time before and after the intervention. (**H**) Brain activation topographic maps at Week 8 for each group, showing the spatial distribution of ΔHbO during the Stroop task. Warmer colors (yellow to red) indicate higher activation levels, whereas cooler colors (blue) indicate lower activation. Across the four groups: control group (MA, *n* = 41), traditional regimen training (TR, *n* = 41), aerobic exercise (AE, *n* = 40), and multimodal cognitive training (MC, *n* = 40). * indicates significant within-group differences between Week 0 and Week 8 (* *p* < 0.05, ** *p* < 0.01). # indicates significant between-group differences at Week 8 (# *p* < 0.05, ## *p* < 0.01). & indicates significant between-group differences at Week 8 (& *p* < 0.05, && *p* < 0.01).

ANOVA was conducted to examine the reaction time and accuracy rate of the Stroop task across four groups (TR, AE, MC, MA) and two test times (pre-test and post-test), as illustrated in Figure 3 and Table 2. For reaction time, the results indicated no significant main effect of time, no significant main effect of group, and no significant time × group interaction. For the accuracy rate, the analysis revealed a significant main effect of time [F (1, 158) = 25.466, *p* < 0.001, η^2^ = 0.139] and a significant time × group interaction [F (3, 158) = 5.011, *p* = 0.002, η^2^ = 0.087]. Post-hoc comparisons showed that at post-test, the accuracy rates of the TR (*p* = 0.006), AE (*p* = 0.024), and MC (*p* < 0.001) groups were significantly higher than their respective pre-test values. In contrast, the MA group showed no significant difference between pre-test and post-test accuracy rates (*p* = 0.82). Additionally, at post-test, the accuracy rate of the MC group was significantly higher than that of the AE group *(p* = 0.01).

### 3.3. Brain Activation Changes Before and After Exercise

Task-related ΔHbO changes in 16 ROIs during the Stroop task were analyzed individually. Significant Time × Group interaction effects were observed in the L-DLPFC, R-DLPFC, and L-FPA, and these effects remained statistically significant after FDR correction for multiple comparisons across the 16 ROIs. The results were displayed in Figure 3 and Table 2. For the L-DLPFC, a significant main effect of time was observed [F (1, 158) = 7.141, *p* = 0.008, η^2^ = 0.043]. A significant time × group interaction was also detected [F (3, 158) = 4.373, *p* = 0.005, η^2^ = 0.077]. For the R-DLPFC, the main effect of time was significant [F (1, 158) = 5.247, *p* = 0.023, η^2^ = 0.032]. The time × group interaction was significant [F (3, 158) = 3.036, *p* = 0.032, η^2^ = 0.055]. For the L-FPA, the main effect of time was significant [F (1, 158) = 6.044, *p* = 0.015, η^2^ = 0.037]. A significant group × time interaction was found [F (3, 158) = 3.816, *p* = 0.011, η^2^ = 0.068]. For the remaining brain regions, neither the main effect of time nor the time × group interaction reached statistical significance. The fNIRS activation topographic maps for all groups were presented in Figure 3H.

The simple effect analysis results indicated that for the L-DLPFC (Figure 3C and Table 2), at post-test, the ΔHbO during the Stroop task in the TR group (*p* = 0.026), AE group (*p* = 0.044), and MC group (*p* = 0.006) was significantly higher compared to pre-test levels. In contrast, there was no statistically significant difference between pre-test and post-test in the MA group. Furthermore, at post-test, there were no significant differences in ΔHbO during the task between the MC and TR groups (*p* = 0.632) or between the MC and AE groups (*p* = 0.634).

For the R-DLPFC (Figure 3D and Table 2), the ΔHbO concentration during the Stroop task in the AE group (*p* = 0.036) and MC group (*p* = 0.014) was significantly higher at post-test compared to pre-test. However, there was no significant difference between pre-test and post-test in the TR and MA groups. Additionally, at post-test, no significant differences in ΔHbO were observed between the MC and TR groups (*p* = 0.098) or between the MC and AE groups (*p* = 0.373).

For the L-FPA (Figure 3E and Table 2), the ΔHbO concentration during the Stroop task in the AE group (*p* = 0.038) and MC group (*p* = 0.002) was significantly higher at post-test compared to pre-test. However, no significant differences were observed between pre-test and post-test in the TR and MA groups. Additionally, at post-test, there were no significant differences in ΔHbO between the MC and TR groups (*p* = 0.234) or between the MC and AE groups (*p* = 0.586).

### 3.4. MES Results Before and After Exercise

ANOVA was conducted on MES-T scores across four groups (TR, AE, MC, MA) and two time points (pre-test and post-test), as shown in Figure 3F and Table 2. The results revealed a significant main effect of time [F (1, 158) = 140.297, *p* < 0.001, η^2^ = 0.470] and a significant time × group interaction [F (3, 158) = 13.833, *p* < 0.001, η^2^ = 0.208]. Further simple effect analysis showed that at pre-test, the average MES-T scores for all groups were below 75, indicating a tendency toward mild cognitive impairment. At post-test, the total scores of the exercise groups increased significantly compared to pre-test scores and exceeded 75, indicating improved cognitive function. For TR (*p* < 0.001, 79.10), AE (*p* < 0.001, 82.88), and MC (*p* < 0.001, 89.40), while there was no statistical difference in the MA before and after the intervention. In addition, there was a significant difference in MES-T between MC and TR (*p* < 0.001), as well as between MC and AE (*p* = 0.002).

### 3.5. Choice Reaction Time Before and After Exercise

ANOVA was conducted on choice reaction time across four groups (TR, AE, MC, MA) and two test times (pre-test and post-test), as shown in Figure 3G and Table 2. The results revealed a significant main effect of time [F (1, 158) = 75.398, *p* < 0.001, η^2^ = 0.323] and a significant time × group interaction [F (3, 158) = 10.931, *p* < 0.001, η^2^ = 0.172]. Further simple effect analysis indicated that at post-test, the choice reaction time in the TR (*p* < 0.001), AE (*p* < 0.001), and MC (*p* < 0.001) groups was significantly shorter than at pre-test. In contrast, there was no statistically significant difference in reaction time between pre-test and post-test in the MA group. Additionally, at post-test, significant differences were observed in reaction time between the MC and TR groups (*p* = 0.001) and between the MC and AE groups (*p* < 0.001).

### 3.6. Behavioral and Brain Activation Level Correlation Analysis of Cognitive Function After Exercise

**Figure 4:**
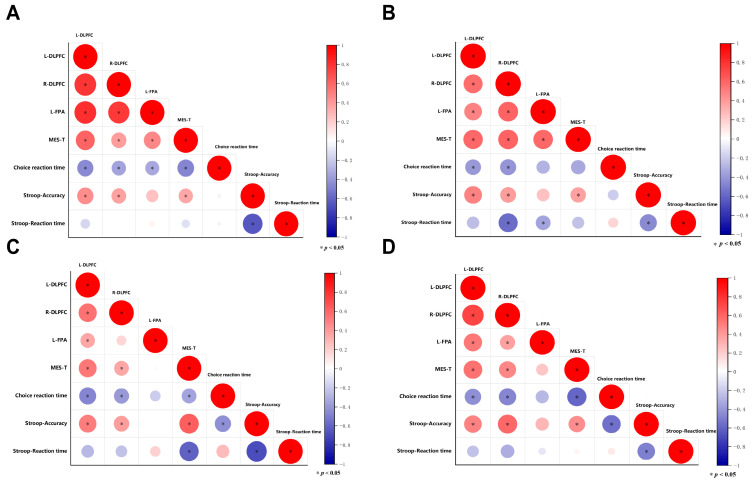
Correlation matrices of brain activity and behavioral performance across the four experimental groups after the intervention. (**A**) MA group, (**B**) TR group, (**C**) AE group, and (**D**) MC group. Each matrix shows Pearson correlation coefficients among regional brain activity, MES-T scores, choice reaction time, Stroop task accuracy, and Stroop task reaction time. Circle size and color indicate the strength and direction of the correlation (red = positive, blue = negative). Statistically significant correlations are indicated in the figure.

Correlation analysis (as shown in Figure 4 and Table 3) revealed that after eight weeks of exercise intervention, Stroop accuracy rates in the MA, TR, AE, and MC groups were significantly positively correlated with ΔHbO in the L-DLPFC and R-DLPFC regions, as well as with MES-T scores. Additionally, MES-T scores in the MA, TR, AE, and MC groups were significantly negatively correlated with choice reaction time.

## 4. Discussion

Methamphetamine-dependent individuals exhibit deficits in processing speed, cognitive flexibility, and working memory compared to healthy individuals [5]. Exercise training effectively enhances cognitive and executive functions in methamphetamine-dependent individuals. In this study, cognitive function was significantly improved following interventions involving traditional regimen training, aerobic exercise, and multimodal cognitive exercise. While there was no notable change in reaction time during the Stroop task, accuracy rates improved significantly. This divergence may reflect differences in task demands. Exercise may improve general response speed and motor processing, which are more directly captured by choice reaction time. In contrast, the Stroop task requires inhibitory control and conflict resolution, and participants may have prioritized accuracy over speed, resulting in improved accuracy without a significant reduction in reaction time. This improvement may be attributed to factors such as increased gray matter volume in the cerebral cortex, enhanced cerebral blood flow, improved brain functional connectivity, and increased neural plasticity [39,40,41]. In addition, methamphetamine use has also been associated with blood–brain barrier dysfunction. Although BBB-related markers were not assessed in the present study, exercise may potentially modulate these processes through improved cerebrovascular regulation and neuroplasticity [20]. Dong conducted a study on the effects of table tennis intervention on the cognitive function of methamphetamine-dependent individuals, reporting significant improvements in Stroop task accuracy alongside reduced reaction times [42]. The discrepancy with the present study may stem from differences in exercise methods and intensity, as cognitive benefits vary by intervention type [43]. Furthermore, this study found that cognitive improvements differed across exercise modalities, with the MC group showing higher Stroop accuracy than the TR group and significantly outperforming the AE group at post-test. This may be related to the multimodal nature of the intervention, which incorporates targeted cognitive training alongside physical activity. By directly activating specific brain regions or altering functional connectivity, this intervention may enhance brain plasticity and further improve the cognitive function of methamphetamine-dependent individuals [39].

The prefrontal cortex plays a critical role in working memory and is essential for cognitive processing. Specifically, the DLPFC is pivotal in regulating cognitive function and is primarily responsible for working memory [44]. Long-term methamphetamine use is associated with cognitive decline or dysfunction, which is closely linked to prefrontal cortex impairment. In this study, after exercise interventions, participants in the AE and MC groups exhibited significant activation in three brain regions: the L-DLPFC, R-DLPFC, and L-FPA. Participants in the TR group showed significant activation only in the L-DLPFC. These findings align with a previous study that used virtual reality competitive bicycles as an intervention for methamphetamine-dependent individuals, demonstrating significant activation in bilateral DLPFC regions post-intervention [13]. Similarly, Song found that during working memory tasks in professional badminton training, the L-FPA and R-DLPFC exhibited greater activation [45]. Increased cerebral blood flow in the prefrontal cortex induced by exercise is believed to be a key mechanism underlying improvements in executive function [46,47].

Research on methamphetamine-dependent individuals has proposed the “functional decline” hypothesis, which highlights the critical role of prefrontal cortex processes. Enhanced activation in specific prefrontal regions is therefore crucial. Within the prefrontal cortex, the L-DLPFC predominantly governs the management of working memory, while the R-DLPFC plays a vital role in reasoning and processing information [48]. The L-FPA, on the other hand, is associated with the execution of complex cognitive tasks [45]. In this study, participants in the AE and MC groups activated more brain regions than those in the TR group. Additionally, the degree of brain activation in the MC group was significantly higher than in the AE group. This finding supports the study by Li [49], which demonstrated through fMRI that Tai Chi combined with cognitive training activated more brain regions compared to cognitive training alone, with Tai Chi enhancing the effects of cognitive training. Based on the results of this study, this may be attributed to the nature of multimodal cognitive training, which combines various forms of physical exercise with cognitive tasks. The synergistic effects of these two components likely amplify each other’s benefits, leading to broader and more significant activation in the brain regions of methamphetamine-dependent individuals.

In the post-test analysis, a significant positive correlation was observed between the accuracy rate of the Stroop task and the activation of both the L-DLPFC and R-DLPFC across all four groups. This finding suggests that participants in the three exercise intervention groups may have experienced activation of the L-DLPFC and R-DLPFC, thereby improving their cognitive function. Activation of the DLPFC plays a critical role in normal decision-making processes [7]. Although both DLPFC and FPA are dominated by ACC, the former is rich in inhibitory neurons while the latter is rich in excitatory neurons, and their roles in cognitive control are not the same [50]. In contrast, the correlation analysis of the L-FPA revealed no significant relationship between L-FPA activation and Stroop task accuracy. This may be because the FPA is typically engaged during more complex multitasking operations [51]. The color-word Stroop task employed in this study likely did not reach the level of complexity required to recruit significant FPA involvement, resulting in relatively low activation of this region. Future research could further investigate the differences in brain region activation by examining methamphetamine-dependent individuals’ performance on cognitive tasks of varying difficulty levels under exercise intervention. This would provide deeper insights into how specific brain regions contribute to cognitive processing and their responsiveness to exercise-based interventions.

In addition to objectively evaluating the intervention outcomes for methamphetamine-dependent individuals, neuropsychological function assessments can also measure treatment effectiveness and serve as a prognostic indicator [52]. MES is a reliable tool for assessing cognitive dysfunction. In this study, the pre-experiment average MES-T scores for all groups were below 75, indicating a tendency toward mild cognitive impairment. Following the intervention, the cognitive function of participants in the three exercise groups improved significantly, with substantial increases in MES-T scores. These findings align with previous research. For example, Jiang demonstrated that acupuncture therapy effectively improved cognitive function in individuals with cognitive impairment [53], with post-intervention MES-T scores significantly higher than pre-intervention scores. Additionally, this study found that the MC group exhibited more significant improvements in MES-T scores compared to the TR and AE groups.

Choice reaction time is an index in China’s “National Physical Fitness Monitoring” system, used to evaluate the coordination of the nervous and muscular systems in adults as well as their rapid reaction ability. It provides valuable insights into the cognitive processing of complex information [54]. Before the intervention, the choice reaction times of the participants in all four groups were below the normal standard for men of the same age, indicating that long-term methamphetamine use leads to declines in cognitive processing and rapid reaction capabilities. After eight weeks of exercise, choice reaction times improved in the three exercise groups, with the MC group showing the most significant improvement. This may be due to the multimodal cognitive training incorporating aerobic, resistance, balance, and coordination exercises, which simultaneously enhance cognitive function and physical coordination.

The correlation analysis further revealed a negative relationship between choice reaction time and MES-T scores. This suggests that improvements in the cognitive function of methamphetamine-dependent individuals correspond to enhanced physical coordination and rapid reaction ability.

## 5. Limitation

This study has several limitations. First, the findings may not be fully generalizable to female methamphetamine-dependent individuals, as only male participants were included. Future studies should include female participants to determine whether the effects of different exercise interventions vary by sex. Second, the intervention period lasted only eight weeks, and no long-term follow-up was conducted. Therefore, the sustainability of the observed cognitive and neural benefits remains unclear. Third, all participants were recruited from a compulsory isolation rehabilitation setting, which may limit the generalizability of the findings to other clinical or community-based populations. In addition, future studies should further explore the relationships among years of methamphetamine use, abstinence duration, cognitive function, and neural activity, particularly in the context of exercise interventions. Such investigations would provide a more comprehensive basis for understanding cognitive recovery and optimizing rehabilitation strategies in methamphetamine-dependent populations.

## 6. Conclusions

Cognitive impairment in methamphetamine-dependent individuals may be associated with reduced prefrontal functional activity in regions such as the L-DLPFC, R-DLPFC, and L-FPA. fNIRS, which enables dynamic monitoring of task-related hemodynamic responses, may be a useful tool for assessing cortical activation associated with cognitive changes in this population. The present study suggests that eight weeks of traditional regimen training, aerobic exercise, and multimodal cognitive exercise training can improve cognitive function in methamphetamine-dependent individuals. These improvements may be associated with activation of the L-DLPFC, R-DLPFC, and L-FPA. Among the three exercise modalities, multimodal cognitive exercise training showed the largest overall benefit in Stroop task accuracy, task-related brain activation, MES-T scores, and choice reaction time. These findings support the potential value of multimodal cognitive exercise training as a promising intervention for cognitive impairment in methamphetamine-dependent populations.

## Figures and Tables

**Table 1:** Demographic, clinical, and intervention-related characteristics of the participants.

Characteristics	MA Group (*n* = 41)	TR Group (*n* = 41)	AE Group (*n* = 40)	MC Group (*n* = 40)	*p*-Value
Age (years)	29.34 ± 6.09	28.61 ± 3.29	30.63 ± 5.00	30.60 ± 4.65	0.16 ^a^
Height (cm)	168.66 ± 5.30	166.63 ± 5.21	167.91 ± 5.58	168.83 ± 6.30	0.278 ^a^
Weight (kg)	69.53 ± 6.94	67.08 ± 8.82	68.11 ± 10.33	70.2 ± 9.35	0.393 ^a^
BMI (kg/m^2^)	24.48 ± 2.66	24.18 ± 3.22	24.14 ± 3.46	24.65 ± 3.12	0.869 ^a^
Mean heart rate during intervention (bpm)	-	124.33 ± 7.39	123.86 ± 7.31	127.67 ± 8.00	0.052 ᵃ
Average daily dose of drug use					0.59 ^b^
<0.1 g	13 (31.7%)	8 (19.5%)	13 (32.5%)	7 (17.5%)	
0.1–0.3 g	15 (36.6%)	13 (31.7%)	20 (50%)	15 (37.5%)	
0.4–1 g	11 (26.8%)	12 (29.3%)	4 (10.0%)	15 (37.5%)	
>1 g	2 (4.9%)	8 (19.5%)	3 (7.5%)	3 (7.5%)	
Frequency of drug use					0.791 ^b^
Once or less per week	20 (48.8%)	22 (53.7%)	27 (67.5%)	21 (52.5%)	
2–5 times a week	13 (31.7%)	12 (29.3%)	6 (15.0%)	12 (30.0%)	
Once or twice a day	5 (12.5%)	4 (9.8%)	3 (7.5%)	5 (12.5%)	
Three or more times a day	3 (7.3%)	3 (7.3%)	4 (10.0%)	2 (5.0%)	
Years of drug use					0.48 ^b^
1–3 years	8 (19.5%)	14 (34.1%)	14 (35.0%)	14 (35.0%)	
4–6 years	12 (29.3%)	7 (17.1%)	10 (25.0%)	9 (22.5%)	
7–9 years	6 (14.6%)	13 (31.7%)	7 (17.5%)	7 (17.5%)	
10–12 years	10 (24.4%)	6 (14.6%)	5 (12.5%)	5 (12.5%)	
13–15 years	2 (4.9%)	1 (2.4%)	3 (7.5%)	2 (5.0%)	
More than 15 years	3 (7.3%)	0 (0%)	1 (2.5%)	3 (7.5%)	

Data are presented as mean ± standard deviation or *n* (%). BMI, body mass index. ^a^ Analyzed by one-way ANOVA. ^b^ Analyzed by χ^2^ test. Mean heart rate during intervention was compared across the three exercise groups only (TR, AE, and MC).

**Table 2:** Results.

Outcome	MA Group (*n* = 41)		TR Group (*n* = 41)		AE Group (*n* = 40)		MC Group (*n* = 40)		Within Group	Between Group	Time × Group Interaction
	Week 0	Week 8	Week 0	Week 8	Week 0	Week 8	Week 0	Week 8	*F*-Value	*F*-Value	*F*-Value
Reaction time in Stroop task	820.4 ± 208.07	823.65 ± 171.57	828.22 ± 166.18	808.67 ± 172.60	747.17 ± 172.20	831.80 ± 199.19	762.81 ± 191.48	735.78 ± 142.81	0.34	2.36	2.09
Accuracy in Stroop task	0.80 ± 0.16	0.79 ± 0.22	0.76 ± 0.24	0.87 ± 0.15	0.74 ± 0.25	0.83 ± 0.22	0.72 ± 0.27	0.93 ± 0.11	25.47 ^##^	0.58	5.01 ^##^
L-DLPFC(ΔHbO) ^a^	−0.00036 ± 0.0064	−0.0025 ± 0.0068	−0.00053 ± 0.0055	0.0022 ± 0.0059	−0.00031 ± 0.0058	0.0022 ± 0.0055	−0.00057 ± 0.0056	0.0029 ± 0.0077	7.14 ^##^	2.66 ^#^	4.37 ^##^
R-DLPFC(ΔHbO) ^b^	−0.00049 ± 0.0081	−0.0024 ± 0.0055	−0.00097 ± 0.0074	0.00086 ± 0.0046	−0.0011 ± 0.0052	0.0020 ± 0.0067	−0.000411 ± 0.0074	0.0031 ± 0.0077	5.24 ^##^	2.285	3.019 ^##^
L-FPA(ΔHbO) ^c^	0.00087 ± 0.0084	−0.0020 ± 0.0086	−0.00087 ± 0.0094	0.0014 ± 0.0058	−0.0016 ± 0.010	0.0027 ± 0.0075	−0.0025 ± 0.011	0.0038 ± 0.013	6.04 ^##^	0.27	3.82 ^##^
MES-T ^d^	70.83 ± 9.40	73.07 ± 10.03	71.85 ± 11.02	79.10 ± 8.69	70.45 ± 13.66	82.88 ± 9.47	73.73 ± 11.30	89.40 ± 8.16	140.30 ^##^	7.84 ^##^	13.83 ^##^
Choice reaction time	0.65 ± 0.07	0.63 ± 0.07	0.63 ± 0.07	0.59 ± 0.08	0.65 ± 0.09	0.61 ± 0.09	0.63 ± 0.07	0.54 ± 0.07	75.40 ^##^	4.44 ^##^	10.93 ^##^

Data are presented as mean ± standard deviation. ^#^ *p* < 0.05. ^##^ *p* < 0.01. ^a^ L-DLPFC, left dorsolateral prefrontal cortex. ^b^ R-DLPFC, right dorsolateral prefrontal cortex. ^c^ L-FPA, left frontopolar area. ^d^ MES-T, total score of the Memory and Executive Screening. ΔHbO values represent task-related relative changes in oxygenated hemoglobin.

**Table 3:** Correlation results.

Group	Variable	L-DLPFC	R-DLPFC	L-FPA	MES-T	Choice Reaction Time	Stroop Accuracy	Stroop Reaction Time
MA	L-DLPFC ^a^	1	0.795 **	0.801 **	0.603 **	−0.442 **	0.449 **	−0.154
	R-DLPFC ^b^	0.795 **	1	0.766 **	0.388 *	−0.354 *	0.369 *	−0.002
	L-FPA ^c^	0.801 **	0.766 **	1	0.477 **	−0.336 *	0.254	0.06
	MES-T ^d^	0.603 **	0.388 *	0.477 **	1	−0.472 **	0.348 *	−0.114
	Choice reaction time	−0.442 **	−0.354 *	−0.336 *	−0.472 **	1	−0.036	−0.029
	Stroop Accuracy	0.449 **	0.369 *	0.254	0.348 *	−0.036	1	−0.660 **
	Stroop Reaction time	−0.154	−0.002	0.06	−0.114	−0.029	−0.660 **	1
TR	L-DLPFC	1	0.563 **	0.485 **	0.588 **	−0.387 *	0.495 **	−0.243
	R-DLPFC	0.563 **	1	0.617 **	0.606 **	−0.403 **	0.392 *	−0.568 **
	L-FPA	0.485 **	0.617 **	1	0.598 **	−0.281	0.255	−0.341 *
	MES-T	0.588 **	0.606 **	0.598 **	1	−0.327 *	0.374 *	−0.233
	Choice reaction time	−0.0387 *	−0.403 **	−0.281	−0.327 *	1	−0.183	0.164
	Stroop Accuracy	0.495 **	0.392 *	0.255	0.374 *	−0.183	1	−0.455 **
	Stroop Reaction time	−0.243	−0.568 **	−0.341 *	−0.233	0.164	−0.455 **	1
AE	L-DLPFC	1	0.549 **	0.345 *	0.528 **	−0.477 **	0.505 **	−0.26
	R-DLPFC	0.549 **	1	0.162	0.358 *	−0.382 *	0.396 *	−0.224
	L-FPA	0.345 *	0.162	1	0.02	−0.187	0.018	0.181
	MES-T	0.528 **	0.358 *	0.02	1	−0.359 *	0.625 **	−0.607 **
	Choice reaction time	−0.477 **	−0.382 *	−0.187	−0.359 *	1	−0.436 **	0.279
	Stroop Accuracy	0.505 **	0.396 *	0.018	0.625 **	−0.436 **	1	−0.683 **
	Stroop Reaction time	−0.26	−0.224	0.181	−0.607 **	0.279	−0.683 **	1
MC	L-DLPFC	1	0.730 **	0.529 **	0.553 **	−0.434 **	0.500 **	−0.223
	R-DLPFC	0.730 **	1	0.364 *	0.467 **	−0.478 **	0.595 **	−0.301
	L-FPA	0.529 **	0.364 *	1	0.231	−0.277	0.286	−0.086
	MES-T	0.553 **	0.467 **	0.231	1	−0.588 **	0.446 **	0.05
	Choice reaction time	−0.434 **	−0.478 **	−0.277	−0.588 **	1	−0.543 **	0.083
	Stroop Accuracy	0.500 **	0.595 **	0.286	0.446 **	−0.543 **	1	−0.492
	Stroop Reaction time	−0.223	−0.301	−0.086	0.05	0.083	−0.492 **	1

* *p* < 0.05. ** *p* < 0.01. ^a^ L-DLPFC, left dorsolateral prefrontal cortex; ^b^ R-DLPFC, right dorsolateral prefrontal cortex; ^c^ L-FPA, left frontopolar area; ^d^ MES-T, total score of the Memory and Executive Screening. fNIRS variables represent task-related ΔHbO.

## Data Availability

The data presented in this study are available on request from the corresponding author due to privacy protection.

## Abbreviations

fNIRS	Functional near-infrared spectroscopy
L-DLPFC	Left dorsolateral prefrontal cortex
R-DLPFC	Right dorsolateral prefrontal cortex
L-FPA	Left frontopolar area
MES	The Memory and Executive Screening
